# Supplementary material for: Stacking Model‐Based Classifiers for Dealing With Multiple Sets of Noisy Labels
Source: Biom J. 2025 Mar 12;67(2):e70042. doi: 10.1002/bimj.70042 (PMC11898607; doi:10.1002/bimj.70042)
Supplement: Supplementary file 1 — Supporting Information [file BIMJ-67-e70042-s001.zip › density-based-ensemble-model-main/competitors_real_data/netlab-master/netlab-master/help/demev2.htm]

Netlab Reference Manual demev2


# demev2

## Purpose

Demonstrate Bayesian classification for the MLP.

## Synopsis

```
demev2
```

## Description

A synthetic two class two-dimensional dataset `x` is sampled
from a mixture of four Gaussians. Each class is
associated with two of the Gaussians so that the optimal decision
boundary is non-linear.
A 2-layer
network with logistic outputs is trained by minimizing the cross-entropy
error function with isotroipc Gaussian regularizer (one hyperparameter for
each of the four standard weight groups), using the scaled
conjugate gradient optimizer. The hyperparameter vectors `alpha` and
`beta` are re-estimated using the function `evidence`. A graph
is plotted of the optimal, regularised, and unregularised decision
boundaries. A further plot of the moderated versus unmoderated contours
is generated.

## See Also

`evidence`, `mlp`, `scg`, `demard`, `demmlp2`

---

**Pages:**
Index


---

Copyright (c) Ian T Nabney (1996-9)
